# Adenosine A_2A_ Receptor Occupancy by Long‐Term Istradefylline Administration in Parkinson's Disease

**DOI:** 10.1002/mds.28378

**Published:** 2020-11-16

**Authors:** Kenji Ishibashi, Yoshiharu Miura, Kei Wagatsuma, Jun Toyohara, Kiichi Ishiwata, Kenji Ishii

**Affiliations:** ^1^ Research Team for Neuroimaging Tokyo Metropolitan Institute of Gerontology Tokyo Japan; ^2^ Department of Neurology Tokyo Metropolitan Cancer and Infectious Diseases Center Komagome Hospital Tokyo Japan; ^3^ Institute of Cyclotron and Drug Discovery Research Southern Tohoku Research Institute for Neuroscience Koriyama Japan; ^4^ Department of Biofunctional Imaging Fukushima Medical University Fukushima Japan

Istradefylline, an adenosine A_2A_ receptor (A_2A_R) antagonist, has been used in Japan since 2013 as an adjunct to levodopa to alleviate *off* episodes in patients with Parkinson's disease (PD) and was approved by the US Food and Drug Administration in 2019.^1^, ^2^ Currently, once‐daily oral administration of 20 or 40 mg is recommended. To understand the pharmacological effects of istradefylline, we measured A_2A_R availability using ^11^C‐preladenant positron emission tomography (PET) before and after single administration of istradefylline in patients with PD and found that occupancy rates of A_2A_Rs in the striatum by single administration of 20 and 40 mg were 39.5% and 52.1%, respectively.^3^ The major drawback of our previous study is that daily administration of istradefylline can increase its baseline plasma concentration, leading to increasing occupancy rates of A_2A_Rs because the plasma elimination half‐life of istradefylline is long (57.09 ± 31.51 hours). The aim of this study was to resolve the drawback of our previous study by recalculating occupancy rates of A_2A_Rs after long‐term administration of istradefylline in patients with PD.

A total of 4 patients with PD aged 78 to 82 years under medication therapy with 2 or more antiparkinsonian drugs including levodopa underwent a total of 2 ^11^C‐preladenant PET to measure A_2A_R availability on 2 occasions: at baseline and more than 2 months after starting daily administration of istradefylline 20 or 40 mg (both n = 2). Thus, the daily dose of istradefylline was set at 20 mg for patients 1 and 2 and 40 mg for patients 3 and 4.

After processing PET images as described previously,^3^ binding potential (BP_ND_) was measured as an index of A_2A_R availability. A_2A_R occupancy upon istradefylline administration was calculated using the following equation: occupancy (%) = 100 × [(BP_ND_ at baseline) – (BP_ND_ at istradefylline‐loading)] / (BP_ND_ at baseline). The relationship between A_2A_R occupancy and dose of istradefylline was modeled using the following equation: occupancy (%) = 100 × [D / (D + ED_50_)], where D refers to dose of istradefylline and ED_50_ refers to the level resulting in 50% receptor occupancy.

The striatal BP_ND_ values at baseline and more than 2 months after starting daily administration of istradefylline were 3.035 and 0.789 in patient 1 (20 mg loading), 2.759 and 0.825 in patient 2 (20 mg loading), 3.259 and 0.359 in patient 3 (40 mg loading), and 3.068 and 0.490 in patient 4 (40 mg loading), as shown in BP_ND_ maps (Fig. 1A–D). The A_2A_R occupancy was 74.0%, 70.1%, 89.0%, and 84.0%, in patients 1, 2, 3, and 4, respectively. The dose‐occupancy curve estimated that ED_50_ was 7.28 mg (Fig. 1E).

**FIG. 1.**
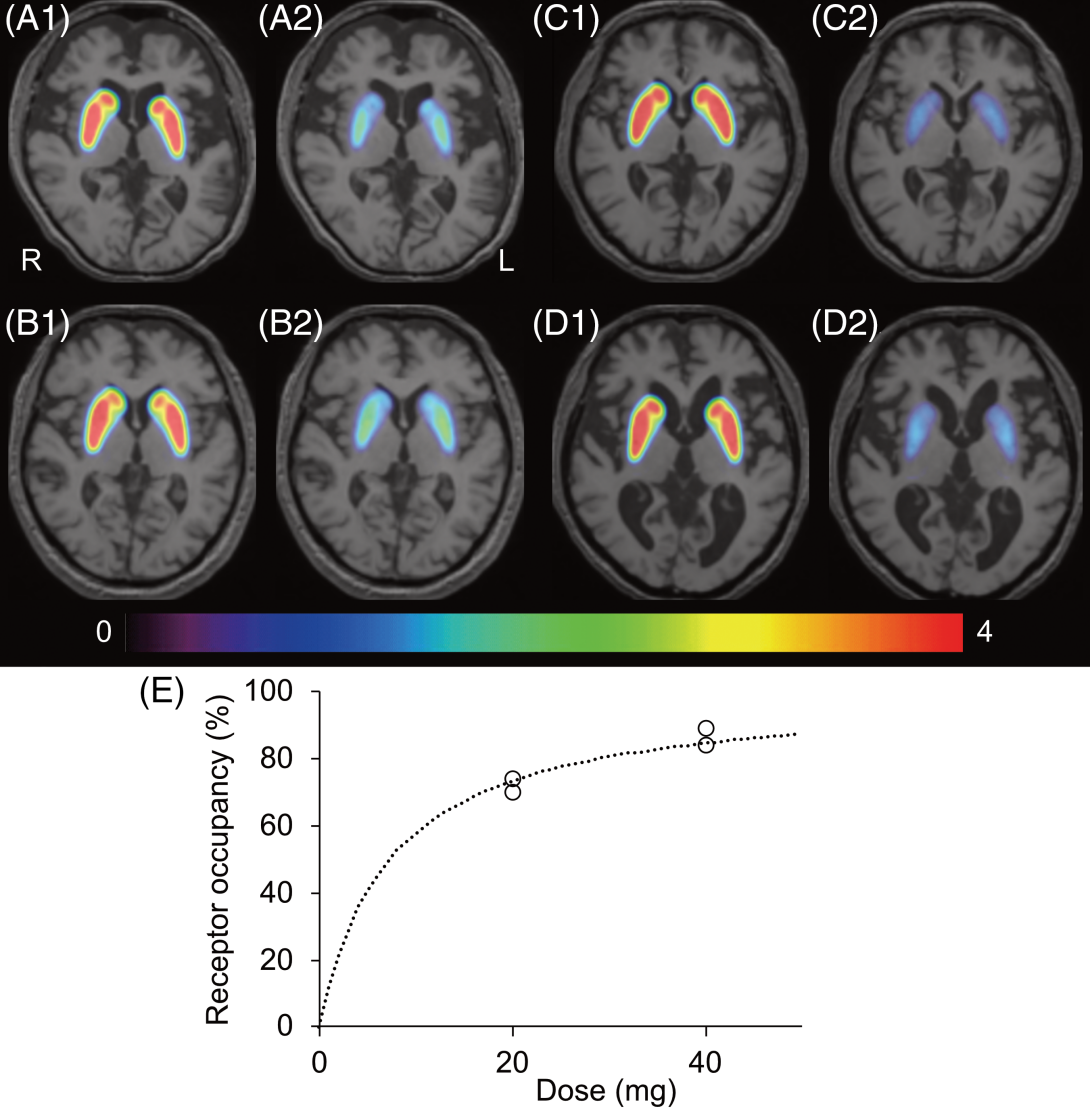
BP_ND_ maps in 4 patients with PD and relationship between adenosine A_2A_ receptor occupancy and dose of istradefylline. BP_ND_ maps of adenosine A_2A_ availability in patients 1 (**A**), 2 (**B**), 3 (**C**), and 4 (**D**) are displayed on structural magnetic resoannce imaging as follows: the baseline (A1, B1, C1, and D1), istradefylline 20 mg loading (A2 and B2), and istradefylline 40 mg loading (C2 and D2). The rainbow‐colored scale represents the magnitude of BP_ND_ values. The dashed curve (**E**) was modeled using the following equation: occupancy (%) = 100 × [D/(D + ED_50_)], where D refers to dose of istradefylline and ED_50_ refers to the level resulting in 50% receptor occupancy. BP_ND_, binding potential; L, left; PD, Parkinson's disease; R, right. [Color figure can be viewed at wileyonlinelibrary.com]

In conclusion, the present study confirmed that istradefylline dose‐dependently binds to A_2A_Rs with doses increasing from 20 to 40 mg in patients with PD under levodopa therapy and found new observations that the mean occupancy rates of A_2A_Rs in the striatum after long‐term administration of istradefylline at 20 and 40 mg doses were 72.1% and 86.5%, respectively, and ED_50_ was 7.28 mg. A more sufficient occupancy of A_2A_Rs can be obtained by long‐term administration of istradefylline than by single administration.

## Author Roles

(1) Research Project: A. Conception, B. Organization, C. Execution; (2) Statistical Analysis: A. Design, B. Execution, C. Review and Critique; (3) Manuscript: A. Writing of the First Draft, B. Review and Critique.

K. Ishibashi: 1A, 1B, 1C, 2A, 2B, 2C, 3A, 3B

Y.M.: 1B, 1C, 2C, 3B

K.W.: 1C, 2C, 3B

J.T.: 1C, 2C, 3B

K. Ishiwata: 1B, 1C, 2C, 3B

K. Ishii: 1B, 1C, 2C, 3B

## Full financial disclosures for the previous 12 months

Nothing to report.
